# A Comparative Study on Root Canal Repair Materials: A Cytocompatibility Assessment in L929 and MG63 Cells

**DOI:** 10.1155/2014/463826

**Published:** 2014-01-12

**Authors:** Yuqing Jiang, Qinghua Zheng, Xuedong Zhou, Yuan Gao, Dingming Huang

**Affiliations:** State Key Laboratory of Oral Diseases Department of Conservative Dentistry and Endodontics, West China Hospital of Stomatology, Sichuan University, Chengdu 610041, China

## Abstract

Cytocompatibility of repair materials plays a significant role in the success of root canal repair. We conducted a comparative study on the cytocompatibility among iRoot BP Plus, iRoot FS, ProRoot MTA, and Super-EBA in L929 cells and MG63 cells. The results revealed that iRoot FS was able to completely solidify within 1 hour. iRoot BP Plus required 7-day incubation, which was much longer than expected (2 hours), to completely set. ProRoot MTA and Super-EBA exhibited a similar setting duration of 12 hours. All the materials except Super-EBA possessed negligible *in vitro* cytotoxicity. iRoot FS had the best cell adhesion capacity in both L929 and MG63 cells. With rapid setting, negligible cytotoxicity, and enhanced cell adhesion capacity, iRoot FS demonstrated great potential in clinical applications. Future work should focus on longer-term *in vitro* cytocompatibility and an *in vivo* assessment.

## 1. Introduction

The selection of the repair material is critical to perform a successful apical root-end surgery or root perforation repair. As a root repair materials, the materials should have excellent characteristics including acceptable biocompatibility, stability in physical and chemical property, radiopacity, set in a wet environment, and good sealing capability. In addition to this traditional concept of the purpose, it has recently been put forward that a root repair material should be able to actively stimulate tissue regeneration, especially after surgical procedures or apical pathosis. The relevant materials should be osteoconductive or osteoinductive [1–3]. So the cytocompatibility of the repair materials plays a significant role in the success of root canal repair [4].

A colorimetric [3-(4,5-dimethyl-thyazol-2-yl)-2,5-diphenyltetrazolium bromide] (MTT) assay is able to determine cellular viability based on the production of a colored formazan compound [5, 6], and this assay kit has been well documented to be a simple and reliable method for the *in vitro* cytotoxicity evaluation of different root canal repair materials [7]. A number of fibroblast cell lines, including L929 and 3T3, have been widely used for MTT assays due to their availability and reproducible outcomes [8–10]. Since there is direct contact between root canal repair materials and periapical tissues, these materials are expected to exhibit osteoinductive or osteoconductive properties that promote bone deposition and eventually root canal repair [4, 11]. Thus, it is also important to assess the cytocompatibility of root canal repair materials on osteoblast-like cells [12].

Various repair materials have been developed for root canal repair. Among these, Super-EBA and mineral trioxide aggregate (MTA) are the most commonly used materials in clinical applications [13–15], though some limitations exist in practice. For example, Super-EBA exhibits cytotoxicity due to the leaching of free eugenol [16–19]. In another variation, MTA is tissue-benign, but the long setting time and the difficulty to maintain the consistency of the material still remain issues [4, 20, 21].

Recently, a series of iRoot materials (iRoot BP Plus and iRoot FS) have emerged as a new generation of root canal repair materials that have a shorter setting duration. These materials are bioceramic-based and the main compositions include calcium silicates and monobasic calcium phosphate, which facilitates the cytocompatibility of these materials [22]. Moreover, previous studies have demonstrated that the contact between osteoblasts and bioceramic components enhances the production of cytokines such as interleukins and tumor necrosis factor [23]. The elevated expression of these bone-resorptive cytokines has a beneficial effect on bone formation [24]. Thus, these materials show high potential for root canal repair.

However, to the best of our knowledge, very few studies have been reported regarding the cytocompatibility and cell-material interaction of these materials. Therefore, the aim of this study was to conduct a comparative assessment on the surface morphology and the cell adhesion capacity of iRoot BP Plus, iRoot FS, ProRoot MTA, and Super-EBA on both fibroblast and osteoblast-like cells models. Furthermore, the time-course *in vitro* cytotoxicity of these materials was assessed.

## 2. Materials and Methods

### 2.1. Materials

The culture medium prepared was Dulbecco's modified Eagle medium (DMEM, Hyclone) supplemented with 10% fetal bovine serum (FBS, Hyclone) and antibiotics (Penicillin 100 U/mL and Streptomycin 100 *μ*g/mL, Gibco). The osteoblast-like cells (MG63) and mouse fibroblast cells (L929) were supplied from State Key Laboratory of Oral Diseases, Sichuan University, China. iRoot BP Plus and iRoot FS were supplied from Innovative Bioceramix Inc. ProRoot MTA was supplied from Dentsply Tulsa Dental. Super-EBA was purchased from Bosworth Co. MTT was purchased from Sigma.

### 2.2. Specimen Preparation

All repair materials (iRoot BP Plus, iRoot FS, ProRoot MTA, and Super-EBA) were prepared under aseptic conditions. iRoot BP Plus and iRoot FS were premixed and packaged in paste forms. ProRoot MTA (in powder form) was mixed with distilled water and Super-EBA was mixed with the working solution supplied by the manufacturer. These materials were placed in sterile custom-made Teflon cylindrical molds (10 mm diameter and 3 mm thickness) at 37°C under 100% humidity. iRoot FS has hardened with a 500 g load by Knoop Hardness Tester (Wilson Instruments, Norwood, MA) after being in the mold for 1 hour. ProRoot MTA and Super-EBA exhibited a similar setting duration of 12 hours. iRoot BP Plus was completely solidified after 7 days.

All the samples were then covered with gauze and immersed in distilled water for solidification at 37°C under 100% humidity for 7 days. The solid materials were subsequently incubated in DMEM following a 1-hour ultraviolet light exposure at 37°C with 5% CO_2_.

### 2.3. Cytotoxicity Assay

The cytotoxicities of these materials were determined as previously described [2, 22]. After 24-hour incubation in DMEM, elutes of each sample (with a surface area to volume ratio of 250 mm^2^/mL) [25] at different time intervals (1, 3, 7, and 14 days) were extracted and filtered through a 0.22 *μ*m filter (Millipore) to remove particulate impurities. These elutes along with their dilutions in DMEM (50% and 25%, resp., without FBS) were subsequently used for cell culture. Fresh DMEM was examined as a control.

Cell suspensions (100 *μ*L/well) were transferred into 96-well plates at a concentration of 5 × 10^4^ cells/well and incubated for 24 hours. Then, the cells were removed and the elutes of different materials (200 *μ*L) were added for another 24-hour incubation period. The relative quantities of cells (optical density (OD) at 490 nm) were evaluated by using a colorimetric (MTT) assay on a microplate reader (Bio-Rad). The relative cell viability was expressed as the ratio of the OD value of elutes at each condition (original elutes and their dilutions) over the control (DMEM).

### 2.4. Surface Morphology and Element Analysis

Each sample was pre-incubated in phosphate buffered saline (PBS) for 2 weeks and the media was refreshed every day. The surface morphology of the prepared samples was examined by using a scanning electron microscope (SEM, Hitachi, S-3000N). The element analysis was performed by a built-in energy dispersive X-ray spectroscope (EDX).

### 2.5. In Vitro Cell Adhesion

Each sample was preincubated in PBS for 2 weeks and the medium was refreshed every day. Cells were loaded onto the samples at a concentration of 5 × 10^4^ cells/sample and allowed to adhere for 24 hours. After incubation, the cell-adhered samples were washed with PBS three times gently and fixed by 2.5% glutaraldehyde for 4 hours. The fixed samples were then treated with a series of graded ethanol solutions (30%, 50%, 70%, 80%, 90%, 95%, and 100%, 15 min each) and then examined by SEM.

## 3. Results

### 3.1. In Vitro Cytotoxicity

**Figure 1:**
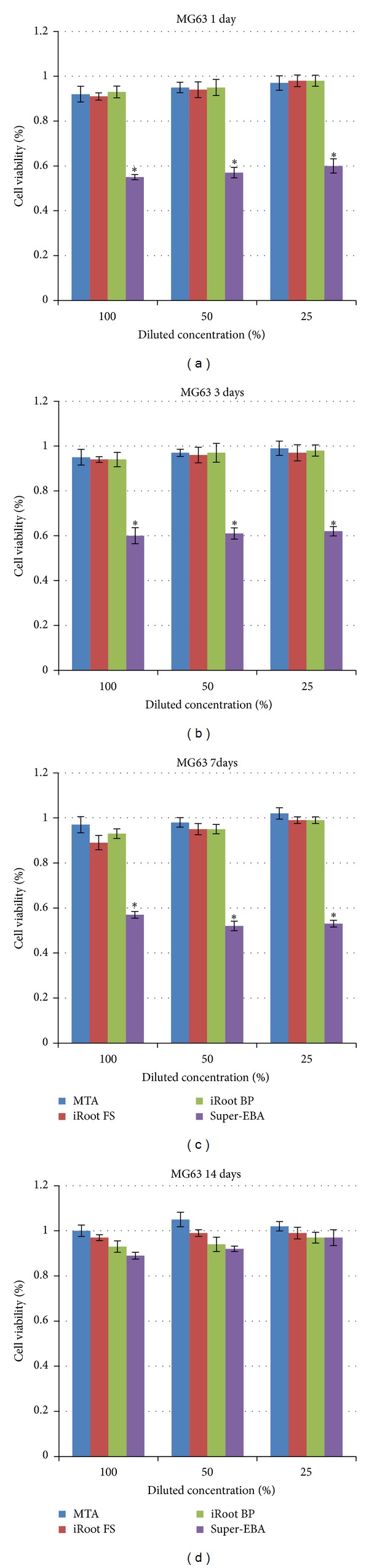
Relative cell viability of different materials determined by MTT assay in MG63 cells upon 1-day (a), 3-day (b), 7-day (c), and 14-day (d) incubation, respectively. *P < 0.05.

**Figure 2:**
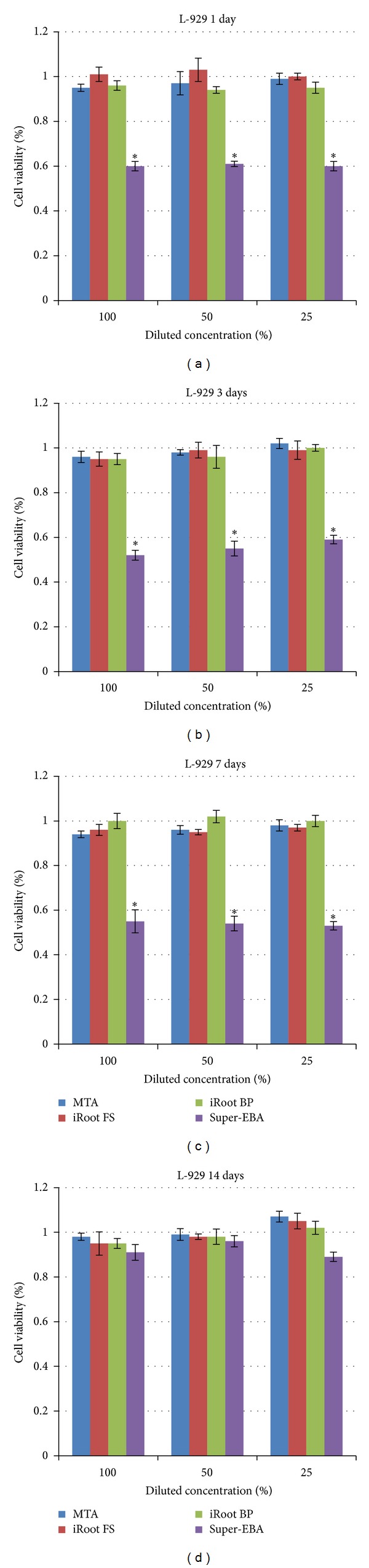
Relative cell viability of different materials determined by MTT assay in L929 cells upon 1-day (a), 3-day (b), 7-day (c), and 14-day (d) incubation, respectively. *P < 0.05.

The *in vitro* cytotoxicities of these materials were compared using the MG63 model (Figure 1) and the L929 model (Figure 2), respectively. The results in the MG63 system were similar to those in the L929 system. In both systems, elutes from the Super-EBA 7-day treatment exhibited relative viabilities less than 60%. The relative viability was dramatically enhanced (90%) by a 14-day culture. Serial dilutions of these elutes had no statistically significant effect on the relative viability. On the other hand, the relative viabilities at different incubation durations (up to 14 days) and different dilutions were all around 95% in the iRoot BP Plus, iRoot FS, and ProRoot MTA groups. These were significantly higher than those of the Super-EBA group.

### 3.2. Surface Morphology and Element Analysis

**Figure 3:**
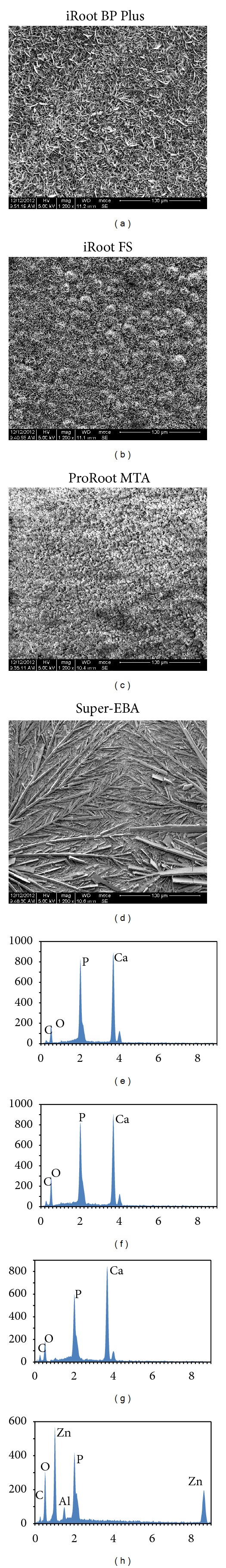
SEM images and corresponding EDX spectrum of iRoot BP Plus ((a) and (e)), iRoot FS ((b) and (f)), ProRoot MTA ((c) and (g)), and Super-EBA ((d) and (h)). The scale bars shown are 100 *μ*m.

The surface morphologies of the materials are illustrated in Figures 3(a)–3(d). iRoot BP Plus and iRoot FS possessed a similar morphology. Schistose and flaky crystals in varied sizes were observed (Figures 3(a) and 3(b)). The average length of flakes was 20 *μ*m for iRoot BP Plus and 5 *μ*m for iRoot FS, respectively. ProRoot MTA showed hexagonal-shaped granules with an average diameter of around 5 *μ*m (Figure 3(c)). The EDX analysis revealed that iRoot BP Plus, iRoot FS, and ProRoot MTA possessed similar element compositions (calcium, carbon, oxygen, and phosphorus). Super-EBA showed a poorly crystallized morphology with large dendrites (~100 *μ*m length), and the main compositions included zinc, aluminum carbon, oxygen, and phosphorus (Figure 3(h)).

### 3.3. Cell Adhesion

**Figure 4:**
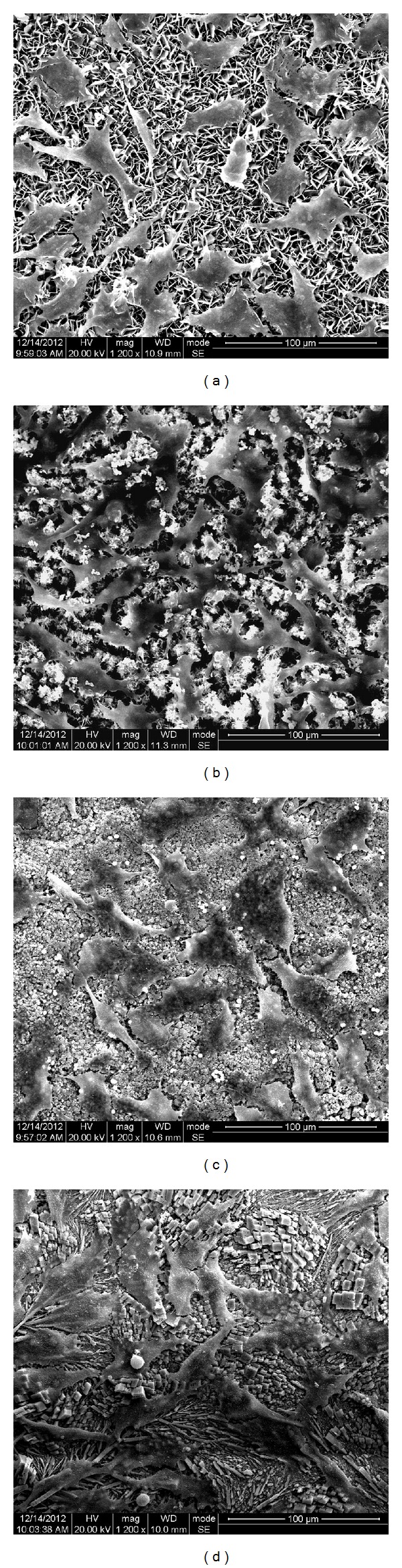
SEM images of iRoot BP Plus (a), iRoot FS (b), ProRoot MTA (c), and Super-EBA (d) after MG63 cell adhesion. The scale bars shown are 100 *μ*m.

As shown in Figure 4, MG63 cells were able to adhere and spread on all the specimens with a characteristic polygonal shape. The iRoot FS group had the highest adhesion density and the highest presence of filopodia (Figure 4(b)) when compared with other specimens.

**Figure 5:**
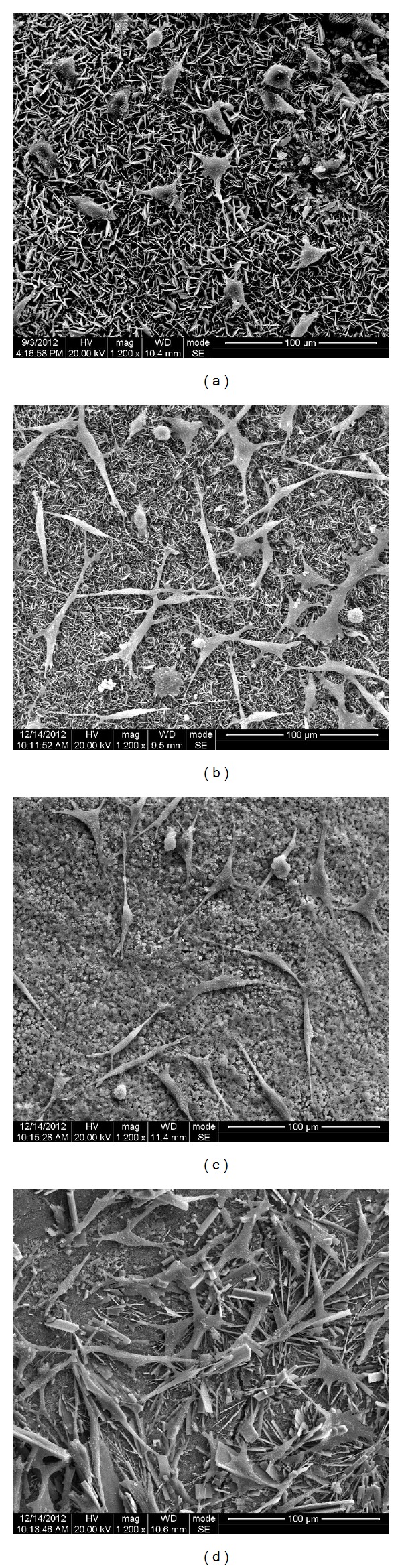
SEM images of iRoot BP Plus (a), iRoot FS (b), ProRoot MTA (c), and Super-EBA (d) after L929 cell adhesion. The scale bars shown are 100 *μ*m.

The adhesion of L929 cells on the specimens is illustrated in Figure 5. After 24-hour incubation, cells on iRoot BP Plus are almost circular, indicating slower attachment and less spread. The cells on the other specimens exhibited a spindle-like shape, which is typical for fibroblasts. The adhesion density in the iRoot FS group was higher than that on other specimens.

## 4. Discussion

iRoot series materials are described as bioceramic materials developed for permanent root canal repair applications with improved handling properties and shorter setting times. They are ready-to-use white hydraulic premixed putty and are composed of calcium silicates, zirconium oxide, tantalum pentoxide, calcium phosphate monobasic, and filler agents. In addition, the manufacturer claims that there is no shrinking during the setting period and that the material is insoluble, radiopaque, and aluminum-free.

The ultimate goal of root canal repair procedure is to permanently seal infected or damaged root canals and to promote the healing of the repair area [1, 26]. For this purpose, a number of materials have been developed and widely used in clinics. In this study, we evaluated four market-available repair materials (iRoot BP Plus, iRoot FS, ProRoot MTA, and Super-EBA). One important criterion of these materials is the setting time [7, 27]. A reduction in setting duration has a beneficial effect on patient relief and reducing bacterial infection [5]. In this study, iRoot FS material was able to completely solidify within one hour at 37°C in 100% relative humidity given an extra load (500 g) to harden the material. Meanwhile, iRoot BP Plus formed a stable structure following seven days of incubation. This was in contrast with the manufacturer's instruction, which indicated that the mixture could be fixed within around 2 hours. A recent study also observed a similar slower setting process (at least 5 days) of iRoot BP Plus [28]. The reason for the delayed setting is still under investigation, but we anticipate that it might be attributed to the humidity-sensitive properties of the compositions. Finally, both ProRoot MTA and Super-EBA required a setting time of at least 12 hours.

The success of root canal therapy relies on the cytocompatibility of the repair materials [22]. In this study, we first examined the cell adhesion capacity of these materials, and the results revealed that iRoot FS exhibited the best cell adhesion (on both L929 and MG63 models) among all the materials. Previous studies demonstrated that cell adhesion was highly dependent on the surface morphology and topography of the materials [29, 30]. Berry et al. indicated that a finer microarchitecture resulted in higher cell attachment and subsequently proliferation rate [29]. These data were in agreement with the results from our current study. Indeed, iRoot BP Plus and iRoot FS possessed a similar microstructure on the surface, with different particle (flake) sizes. More cells (both L929 and MG63) were attached on materials with smaller particle sizes (iRoot FS).

Moreover, in this study, the *in vitro* cytotoxicities of these materials were evaluated by using an MTT assay. The cytotoxicity of the materials could be caused by the presence of toxic components or soluble materials that leach into the surrounding fluids in the bony crypt [17]. Our results revealed that Super-EBA exhibited a significantly higher cytotoxicity than the other materials upon *in vitro* culture. Although Super-EBA has been widely used in clinical practice, the cytotoxicity remains an issue, and recent studies have demonstrated that the cytotoxicity is mainly induced by the leaching of free eugenol [13]. On the other hand, in this study, iRoot BP plus, iRoot FS, and ProRoot MTA exhibited negligible cytotoxicity. This can be explained by the nontoxic components, including calcium and phosphorus, of these materials. Furthermore, the presence of bioceramic facilitated the formation of a hydroxyapatite or apatite-like layer (biomineralization), which further stabilizes the structure and prevents the overdose of component leaching [31–33]. These results are in agreement with previous studies [22].

Taken together, iRoot FS demonstrated great potential in further clinical applications due to its rapid setting, negligible cytotoxicity, and enhanced cell adhesion capacity compared with other commonly used root canal repair materials (Table 1).

## 5. Conclusion

A comparative study was conducted on four root canal repair materials by using both L929 and MG63 cells. The results demonstrated that iRoot FS exhibited the best cell adhesion capacity, and only Super-EBA possessed *in vitro* cytotoxicity. Given the rapid solidification (within one hour) of iRoot FS, this material showed high potential for further clinical applications. Future work should focus on long-term *in vitro* cytotoxicity and an *in vivo* assessment.

## Figures and Tables

**Table 1:** Characteristics of different root canal repair materials.

Specimen	Initial form	Setting time	Surface morphology	Particle size
iRoot BP Plus	Premixed paste	7 days*	Schistose and flaky crystals	20 *μ*m
iRoot FS	Premixed paste	1 hour	Schistose and flaky crystals	5 *μ*m
ProRoot MTA	Powder	4 hours	Hexagonal granules	5 *μ*m
Super-EBA	Powder	12 hours	Dendrites	100 *μ*m

*Note: the expected setting time of iRoot BP Plus is around 2 hours according to manufacturer's instruction.
